# Supplementary material for: Biotin methyl ester enhances cargo release in RUSH system and enables rapid biotinylation with TurboID
Source: Commun Biol. 2025 Dec 16;8:1767. doi: 10.1038/s42003-025-09176-4 (PMC12708715; doi:10.1038/s42003-025-09176-4)
Supplement: Supplementary file 2 — Supplementary Information [file 42003_2025_9176_MOESM2_ESM.pdf]

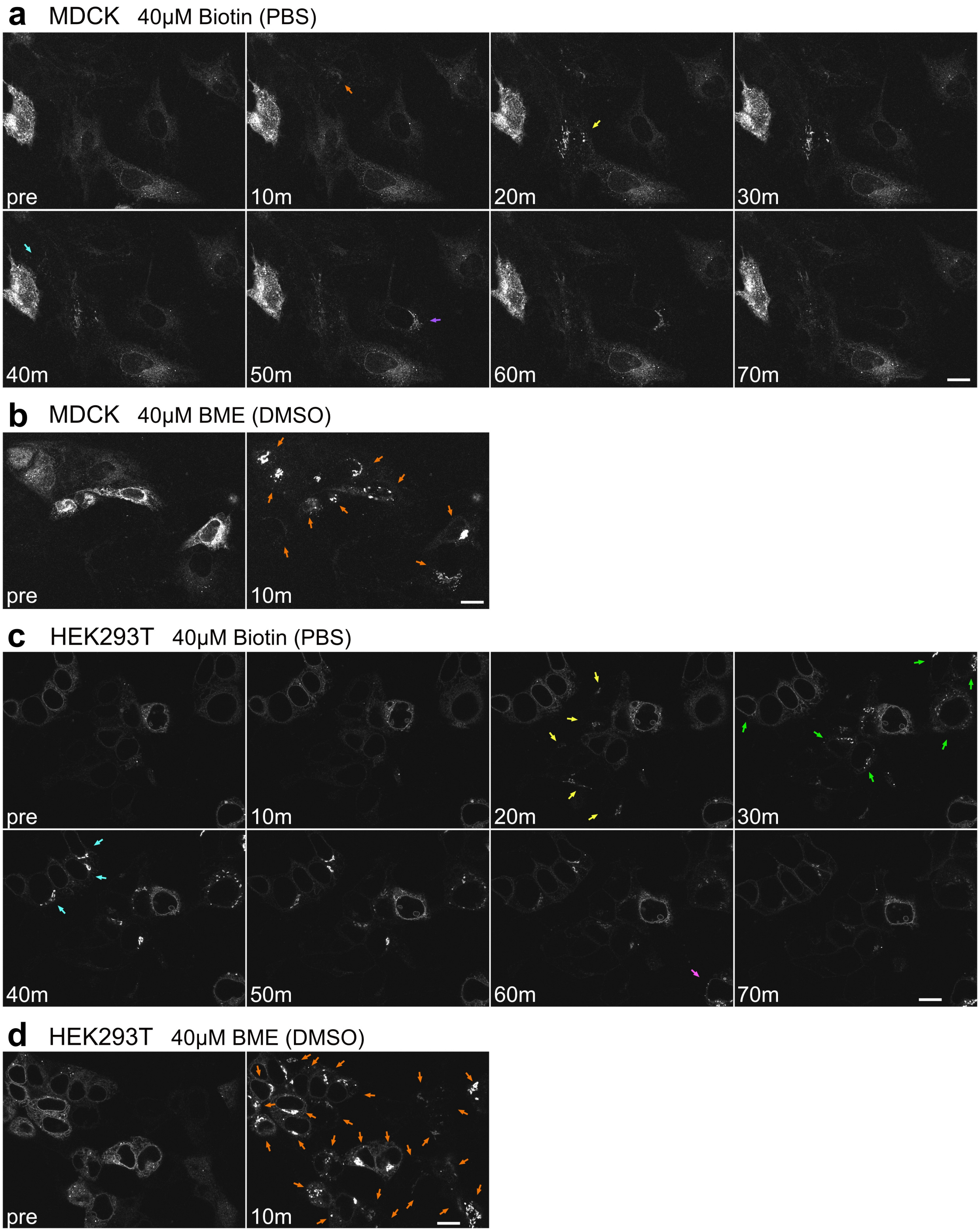
a MDCK 40μM Biotin (PBS)
pre
10m
20m
30m
40m
50m
60m
70m
b MDCK 40μM BME (DMSO)
pre
10m
c HEK293T 40μM Biotin (PBS)
pre
10m
20m
30m
40m
50m
60m
70m
d HEK293T 40μM BME (DMSO)
pre
10m

Supplementary Figure 1 Synchronous release of cargos in the RUSH system by BME in MDCK and HEK293T cells

a, c SBP::GFP::GPI localizations before (upper left) and at 10, 20, 30, 40, 50, 60, and 70 min after addition of 40 μM biotin in the RUSH system in MDCK (**a**) or HEK293T cells (**c**). Arrows indicate cells in which the SBP::GFP::GPI cargo started to accumulate in the Golgi apparatus at that time point.

b, d SBP::GFP::GPI localizations before (left) and at 10 min after addition of 40 μM BME in the RUSH system in MDCK (**b**) or HEK293T cells (**d**). Arrows indicate cells in which the SBP::GFP::GPI cargo began to accumulate in the Golgi apparatus.

Scale bars: 20 μm (**a**–**d**).

BME, biotin methyl ester; RUSH, retention using selective hooks.

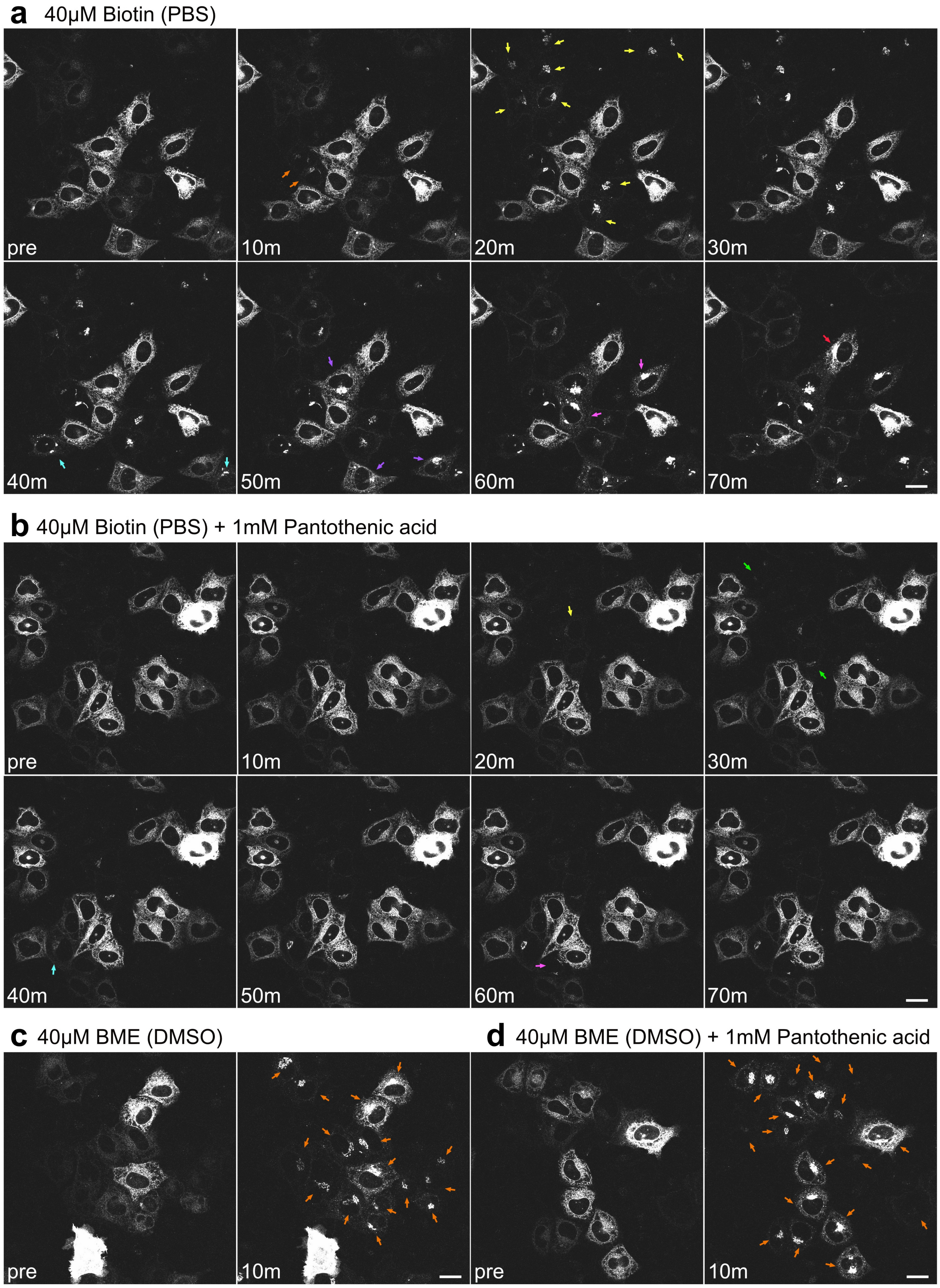
a 40μM Biotin (PBS)
pre
10m
20m
30m
40m
50m
60m
70m
b 40μM Biotin (PBS) + 1mM Pantothenic acid
pre
10m
20m
30m
40m
50m
60m
70m
c 40μM BME (DMSO)
pre
10m
d 40μM BME (DMSO) + 1mM Pantothenic acid
pre
10m

Supplementary Figure 2 Synchronous release of cargos in the RUSH system by BME without SMVT activity in HeLa cells.

a, b SBP::GFP::GPI localizations before (upper left) and at 10, 20, 30, 40, 50, 60, and 70 min after addition of 40 µM biotin in the RUSH system in HeLa cells with (**b**) or without (**a**) 1mM pantothenic acid, an SMVT inhibitor. Arrows indicate cells in which the SBP::GFP::GPI cargo started to accumulate in the Golgi apparatus at that time point.

c, d SBP::GFP::GPI localizations before (left) and at 10 min after addition of 40 µM BME in the RUSH system in HeLa cells with (**d**) or without (**c**) 1 mM pantothenic acid, an SMVT inhibitor. Arrows indicate cells in which the SBP::GFP::GPI cargo began to accumulate in the Golgi apparatus.

Scale bars: 20 µm (**a**–**d**).

BME, biotin methyl ester; RUSH, retention using selective hooks.

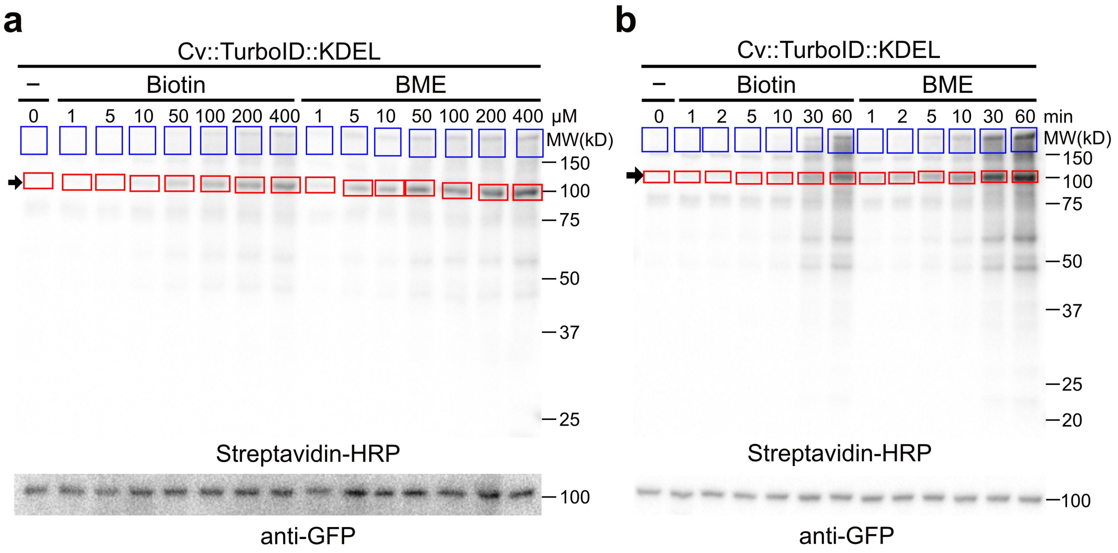

Supplementary Figure 3 Areas to be used for immunoblotting membrane plots

a, b Immunoblotting membranes for detecting biotinylated proteins (top) and Cv::TurboID::KDEL (bottom). Red and blue rectangles indicate the areas used to plot the relative amounts of Cv::TurboID::KDEL self-biotinylation (red) and biotinylation (blue) by Cv::TurboID::KDEL.

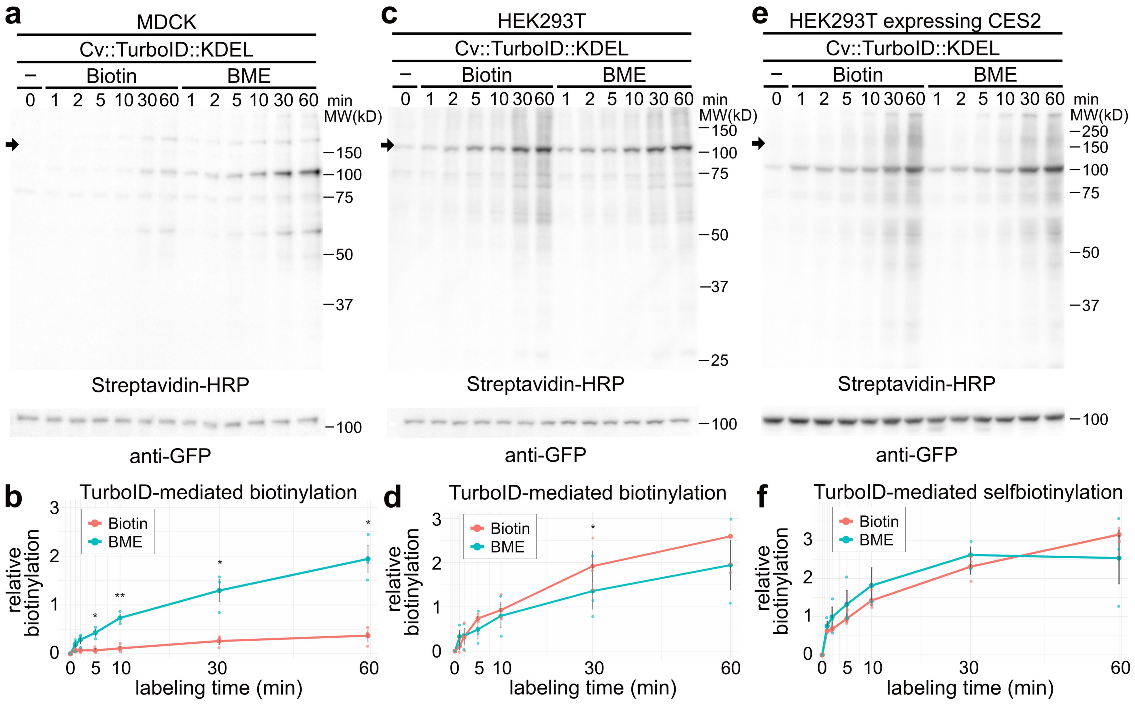
a
MDCK
Cv::TurboID::KDEL
–
Biotin
BME
0 1 2 5 10 30 60 1 2 5 10 30 60 min
MW(kD)
150
100
75
50
37
Streptavidin-HRP
100
anti-GFP
b
TurboID-mediated biotinylation
Biotin
BME
relative biotinylation
labeling time (min)
c
HEK293T
Cv::TurboID::KDEL
–
Biotin
BME
0 1 2 5 10 30 60 1 2 5 10 30 60 min
MW(kD)
150
100
75
50
37
25
Streptavidin-HRP
100
anti-GFP
d
TurboID-mediated biotinylation
Biotin
BME
relative biotinylation
labeling time (min)
e
HEK293T expressing CES2
Cv::TurboID::KDEL
–
Biotin
BME
0 1 2 5 10 30 60 1 2 5 10 30 60 min
MW(kD)
250
150
100
75
50
37
Streptavidin-HRP
100
anti-GFP
f
TurboID-mediated selfbiotinylation
Biotin
BME
relative biotinylation
labeling time (min)

Supplementary Figure 4 BME enables rapid biotinylation with TurboID in MDCK and HEK293T cells

MDCK (a, b), HEK293T (c, d), and HEK293T cells expressing CES2 (e, f) were used for this study.

a, c, e Detection of biotinylated proteins by immunoblotting. Extracts from cells expressing Cv::Turbo::KDEL were prepared after 0, 1, 2, 5, 10, 30, and 60 min incubation with biotin or BME at 50 μM. Streptavidin-HRP or anti-GFP antibodies were used to detect biotinylated proteins (top) or Cv::TurboID::KDEL (bottom), respectively. The arrow indicates the band for Cv::TurboID::KDEL.

b, d, f Relative amounts of self-biotinylation. The intensity of Cv::TurboID::KDEL (red rectangles in Fig. S5a, b, c) was measured in three independent experiments. Significance was determined using a two-tailed paired Student's *t*-test. Exact P values are 0.222 (1), 0.125 (2), 0.018 (5), 0.001 (10), 0.022 (30), and 0.011 (60) in **b**, 0.428 (1), 0.946 (2), 0.443 (5), 0.488 (10), 0.019 (30), and 0.119 (60) in **d**, and 0.385 (1), 0.344 (2), 0.406 (5), 0.491 (10), 0.343 (30), and 0.457 (60) in **f**. Error bars are presented in **b**, **d**, and **f** as mean ± SE. Significance was determined using a two-tailed paired Student's *t*-test: *$p < 0.05$, and **$p < 0.01$.

BME, biotin methyl ester.

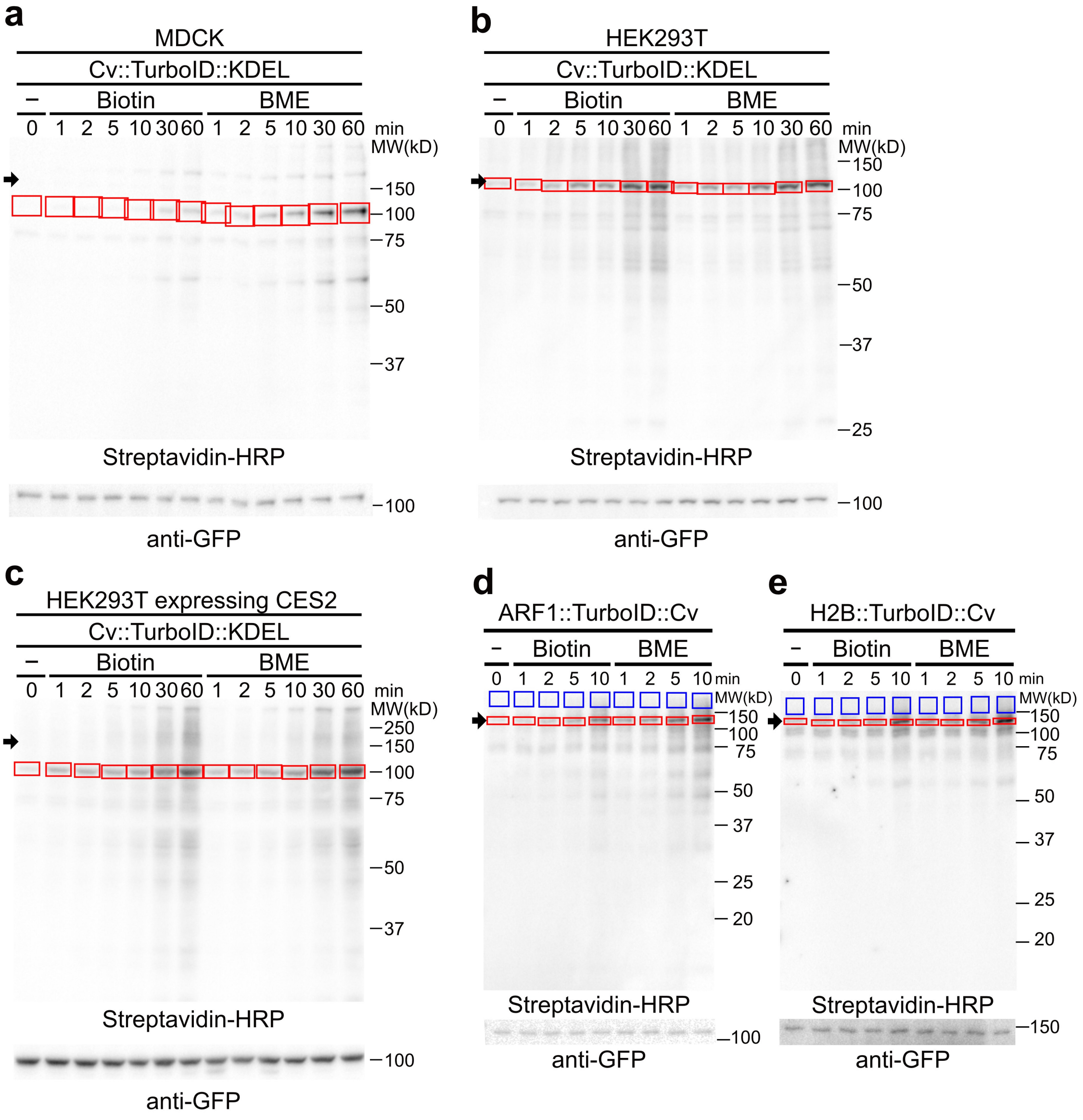
a
MDCK
Cv::TurboID::KDEL
– Biotin BME
0 1 2 5 10 30 60 1 2 5 10 30 60 min
MW(kD)
150
100
75
50
37
Streptavidin-HRP
100
anti-GFP
b
HEK293T
Cv::TurboID::KDEL
– Biotin BME
0 1 2 5 10 30 60 1 2 5 10 30 60 min
MW(kD)
150
100
75
50
37
25
Streptavidin-HRP
100
anti-GFP
c
HEK293T expressing CES2
Cv::TurboID::KDEL
– Biotin BME
0 1 2 5 10 30 60 1 2 5 10 30 60 min
MW(kD)
250
150
100
75
50
37
Streptavidin-HRP
100
anti-GFP
d
ARF1::TurboID::Cv
– Biotin BME
0 1 2 5 10 1 2 5 10 min
MW(kD)
150
100
75
50
37
25
20
Streptavidin-HRP
100
anti-GFP
e
H2B::TurboID::Cv
– Biotin BME
0 1 2 5 10 1 2 5 10 min
MW(kD)
150
100
75
50
37
25
20
Streptavidin-HRP
150
anti-GFP

Supplementary Figure 5 Areas to be used for immunoblotting membrane plots

MDCK cells (a), HEK293T cells (b), HEK293T cells expressing CES2 (c), and HeLa cells (d and e).

a–**c** Immunoblotting membranes used to detect biotinylated proteins (top) and Cv::TurboID::KDEL (bottom). Red rectangles indicate the areas used to plot the relative amounts of Cv::TurboID::KDEL self-biotinylation.

d, e Immunoblotting membranes used to detect biotinylated proteins (top) and ARF1::TurboID::Cv (**d**) or H2B::TurboID::Cv (**e**) (bottom). The red and blue rectangles indicate the areas used to plot the relative amounts of self-biotinylation (red) and to plot the relative amounts of biotinylation (blue).

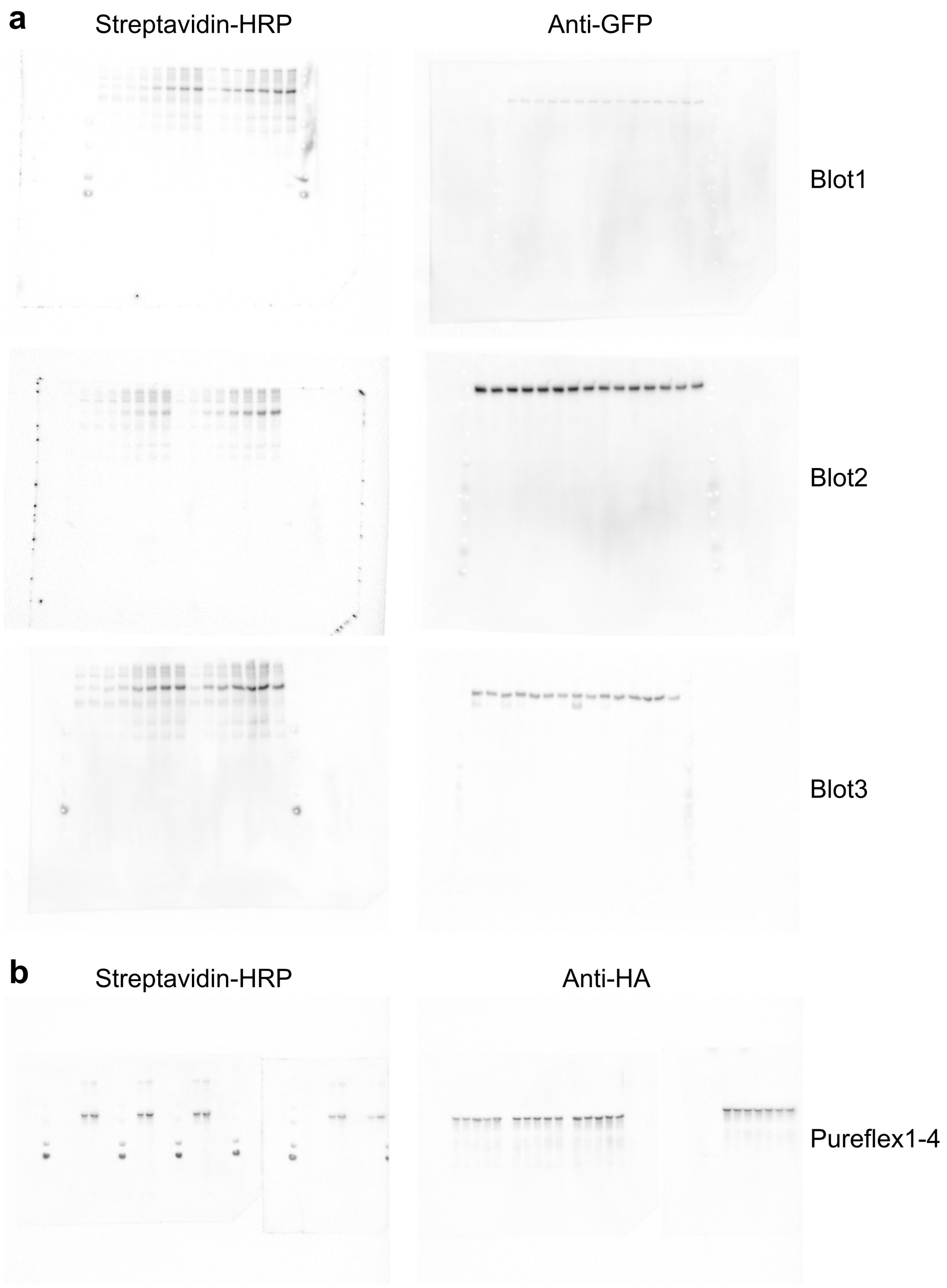

Supplementary Figure 6

a Original immunoblotting membranes used for Figure 2c-e.

b Original immunoblotting membranes used for Figure 2f, g.

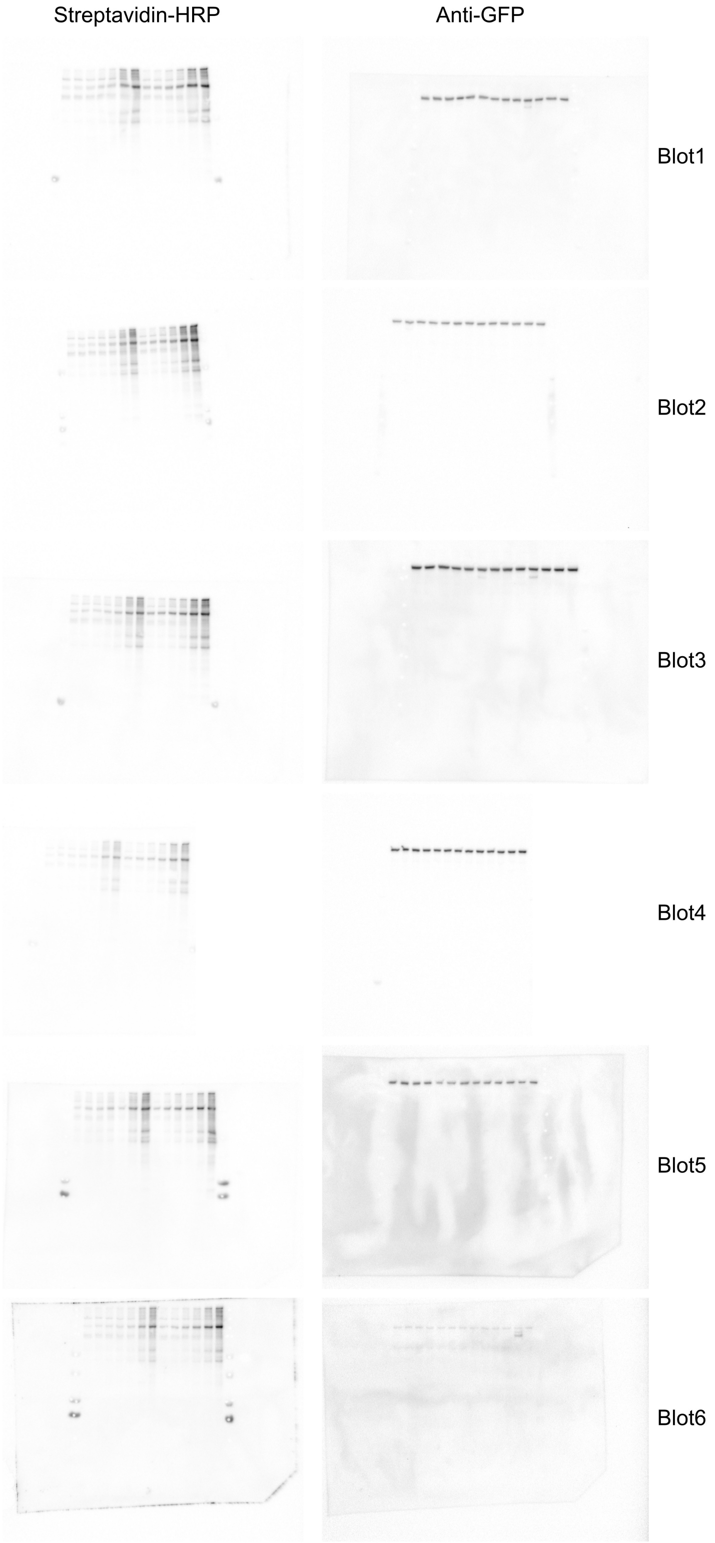

Supplementary Figure 7

Original immunoblotting membranes used for Figure 3 c-e.

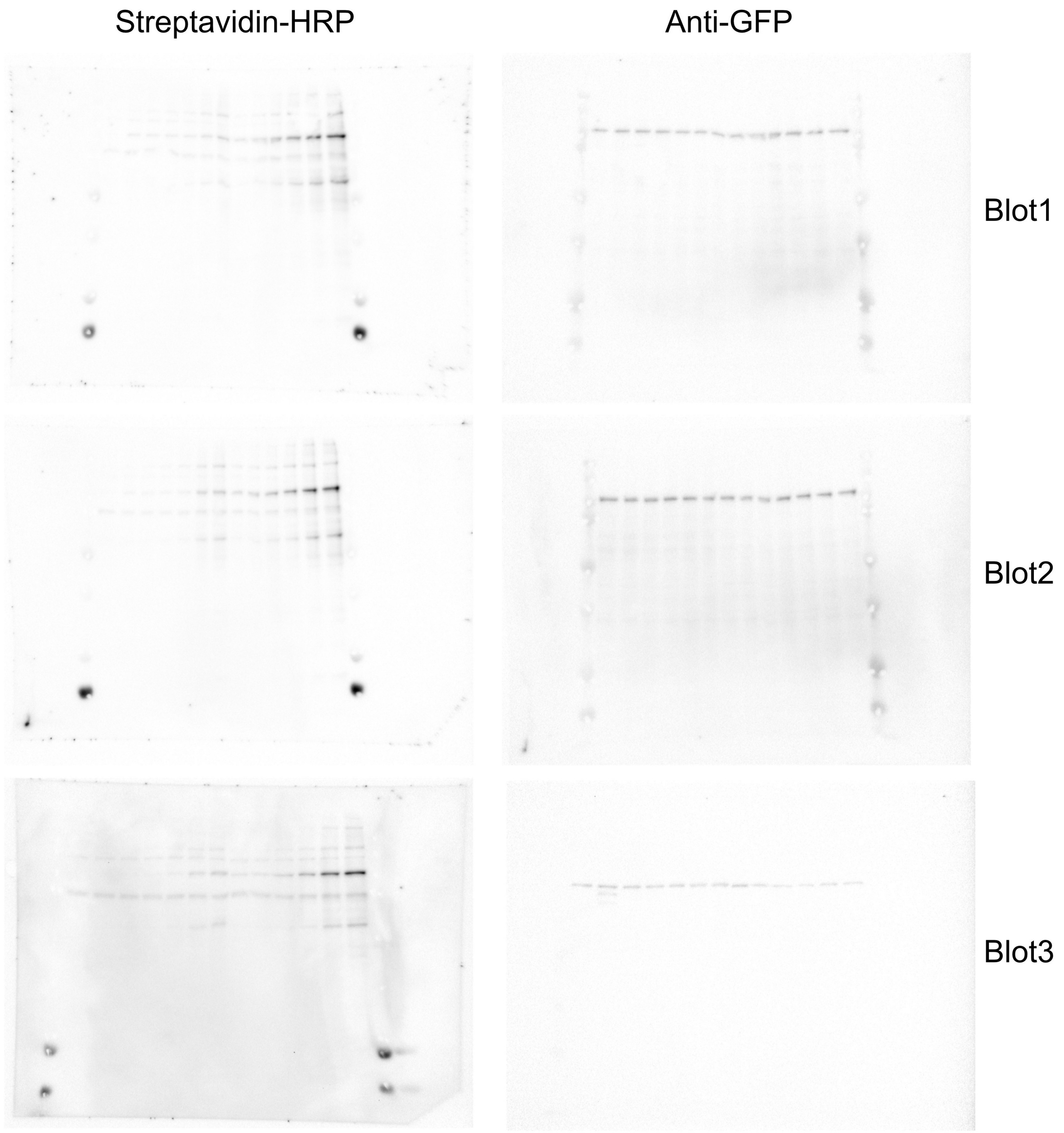

Supplementary Figure 8

Original immunoblotting membranes used for Supplementary Figure S4a, b.

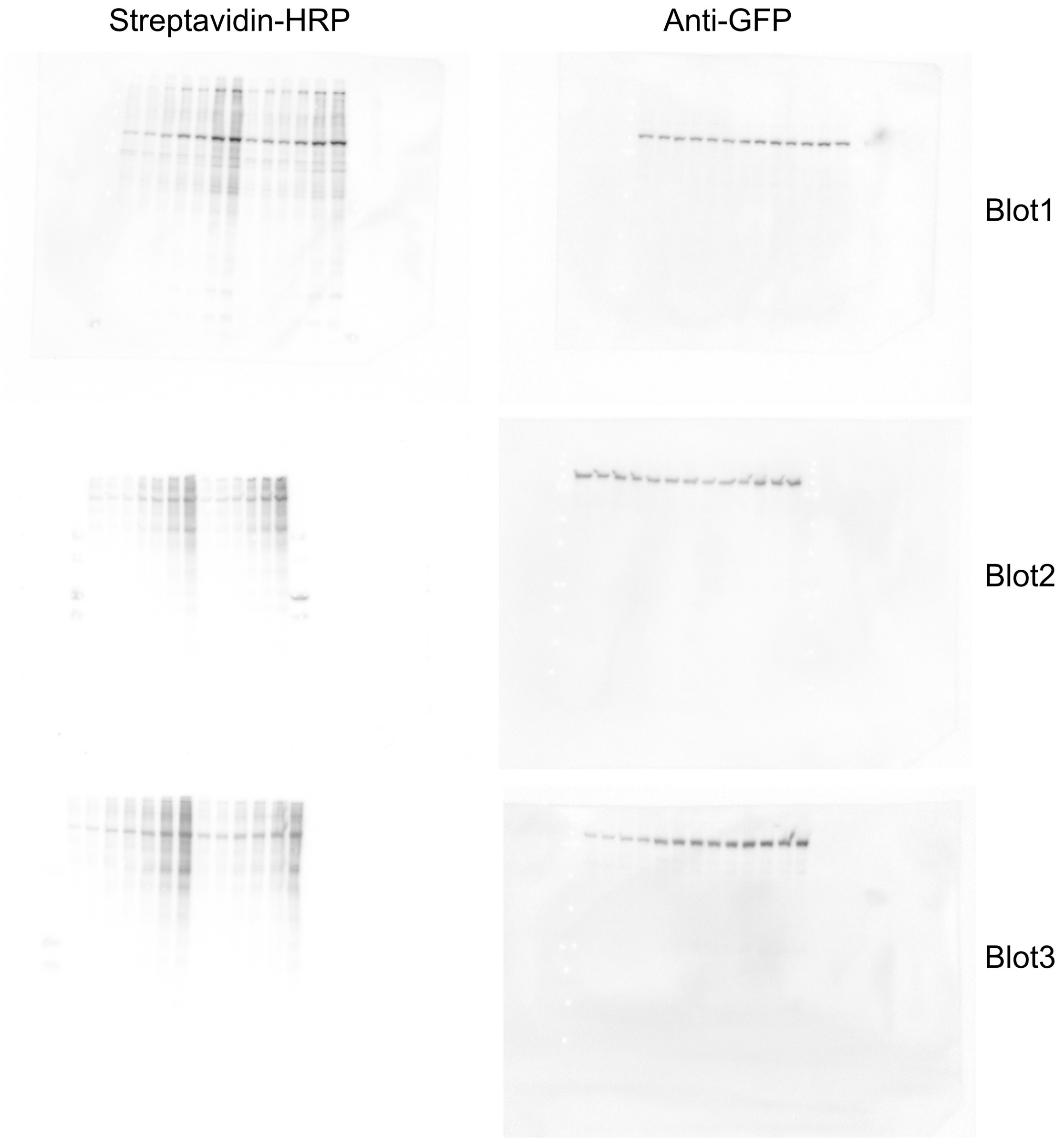

Supplementary Figure 9

Original immunoblotting membranes used for Supplementary Figure S4c, d.

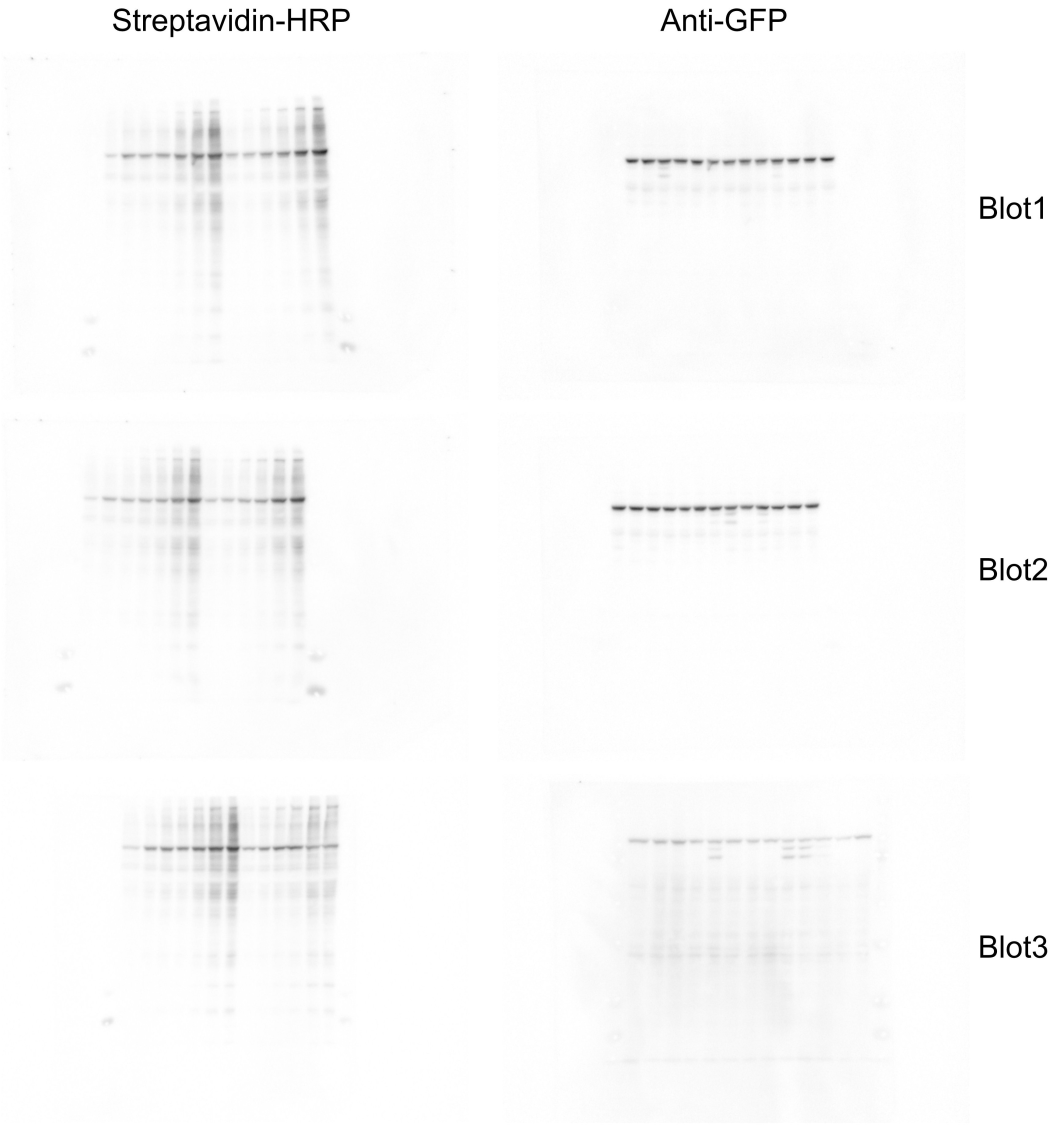

Supplementary Figure 10

Original immunoblotting membranes used for Supplementary Figure S4e, f.

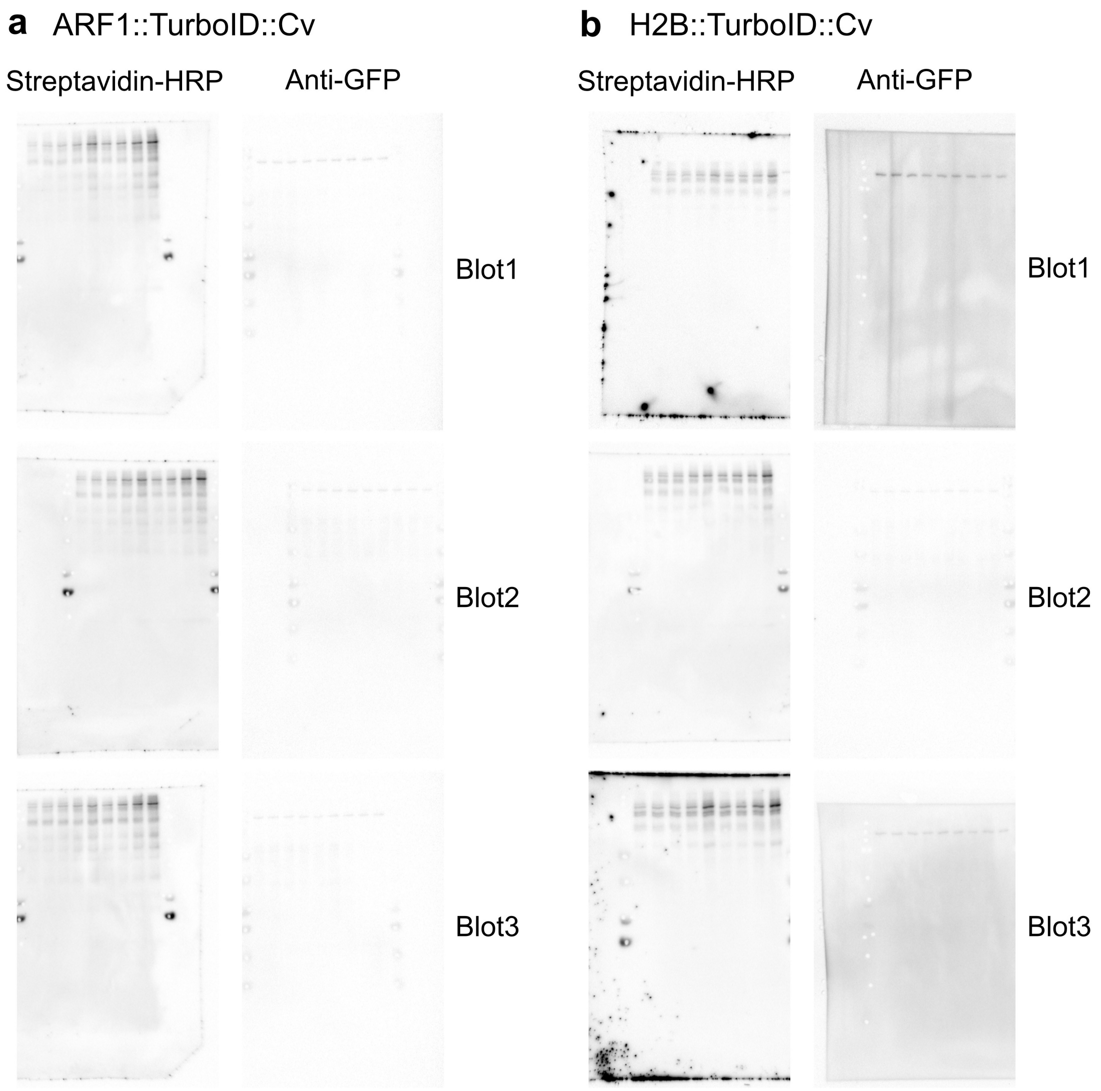

Supplementary Figure 11

a Original immunoblotting membranes used for Supplementary Figure 4c, e, g.

b Original immunoblotting membranes used for Supplementary Figure 4d, f, h.
