# Supplementary material for: Biotin methyl ester enhances cargo release in RUSH system and enables rapid biotinylation with TurboID
Source: Commun Biol. 2025 Dec 16;8:1767. doi: 10.1038/s42003-025-09176-4 (PMC12708715; doi:10.1038/s42003-025-09176-4)
Supplement: Supplementary file 3 — Description of Additional Supplementary Files [file 42003_2025_9176_MOESM3_ESM.pdf]

Description of Additional Supplementary Files

File name: Supplementary Data
Description: Source data for Figures

File Name: Supplementary Video 1
Description:
Video of live imaging observation of SBP::GFP::GPI transport in HeLa cells using the RUSH system.
Live imaging observation of SBP::GFP::GPI transport at 5-min intervals initiated by 40 µM biotin (left) and 40 µM BME (right) in the RUSH system. SBP::GFP::GPI is green and GalT::iRFP713 is magenta.
Scale bars: 20 µm.

File Name: Supplementary Video 2
Description:
Video of live imaging observation of SBP::GFP::GPI transport in MDCK cells using the RUSH system.
Live imaging observation of SBP::GFP::GPI transport at 5-min intervals initiated by 40 µM biotin (left) and 40 µM BME (right) in the RUSH system. SBP::GFP::GPI is green and GalT::iRFP680 is magenta.
Scale bars: 20 µm.

File Name: Supplementary Video 3
Description:
Video of live imaging observation of SBP::GFP::GPI transport in HEK293T cells using the RUSH system.
Live imaging observation of SBP::GFP::GPI transport at 5-min intervals initiated by 40 µM biotin (left) and 40 µM BME (right) in the RUSH system. SBP::GFP::GPI is green and GalT::iRFP680 is magenta.
Scale bars: 20 µm.

File Name: Supplementary Video 4
Description:
Video of live imaging observation of biotin induced SBP::GFP::GPI transport in HeLa cells using the RUSH system with and without pantothenic acid.
Live imaging observation of SBP::GFP::GPI transport at 10-min intervals initiated by 40 µM biotin with (right) and without pantothenic acid (left) in the RUSH system. SBP::GFP::GPI is green and Ruby::GM130 is magenta.
Scale bars: 20 µm.

File Name: Supplementary Video 5
Description:

Video of live imaging observation of BME induced SBP::GFP::GPI transport in HeLa cells using the RUSH system with and without pantothenic acid.

Live imaging observation of SBP::GFP::GPI transport at 10-min intervals initiated by 40 µM BME with (right) and without pantothenic acid (left) in the RUSH system. SBP::GFP::GPI is green and Ruby::GM130 is magenta.

Scale bars: 20 µm.
