# Supplementary material for: Biotin methyl ester enhances cargo release in RUSH system and enables rapid biotinylation with TurboID
Source: Commun Biol. 2025 Dec 16;8:1767. doi: 10.1038/s42003-025-09176-4 (PMC12708715; doi:10.1038/s42003-025-09176-4)
Supplement: Supplementary file 10 — Reporting Summary [file 42003_2025_9176_MOESM10_ESM.pdf]

nature portfolio

Corresponding author(s): Akiko K satoh

Last updated by author(s): 2015 04 08

Reporting Summary

Nature Portfolio wishes to improve the reproducibility of the work that we publish. This form provides structure for consistency and transparency in reporting. For further information on Nature Portfolio policies, see our Editorial Policies and the Editorial Policy Checklist.

Statistics

For all statistical analyses, confirm that the following items are present in the figure legend, table legend, main text, or Methods section.

n/a	Confirmed	
☐	☒	The exact sample size (*n*) for each experimental group/condition, given as a discrete number and unit of measurement
☐	☒	A statement on whether measurements were taken from distinct samples or whether the same sample was measured repeatedly
☐	☒	The statistical test(s) used AND whether they are one- or two-sided<br>*Only common tests should be described solely by name; describe more complex techniques in the Methods section.*
☒	☐	A description of all covariates tested
☐	☒	A description of any assumptions or corrections, such as tests of normality and adjustment for multiple comparisons
☐	☒	A full description of the statistical parameters including central tendency (e.g. means) or other basic estimates (e.g. regression coefficient) AND variation (e.g. standard deviation) or associated estimates of uncertainty (e.g. confidence intervals)
☐	☒	For null hypothesis testing, the test statistic (e.g. *F*, *t*, *r*) with confidence intervals, effect sizes, degrees of freedom and *P* value noted<br>*Give P values as exact values whenever suitable.*
☒	☐	For Bayesian analysis, information on the choice of priors and Markov chain Monte Carlo settings
☒	☐	For hierarchical and complex designs, identification of the appropriate level for tests and full reporting of outcomes
☒	☐	Estimates of effect sizes (e.g. Cohen's *d*, Pearson's *r*), indicating how they were calculated

Our web collection on statistics for biologists contains articles on many of the points above.

Software and code

Policy information about availability of computer code

Data collection	We did not use any software and codes.
Data analysis	R (4.3.1) and Fiji (2.9.0)

For manuscripts utilizing custom algorithms or software that are central to the research but not yet described in published literature, software must be made available to editors and reviewers. We strongly encourage code deposition in a community repository (e.g. GitHub). See the Nature Portfolio guidelines for submitting code & software for further information.

Data

Policy information about availability of data

All manuscripts must include a data availability statement. This statement should provide the following information, where applicable:

- Accession codes, unique identifiers, or web links for publicly available datasets
- A description of any restrictions on data availability
- For clinical datasets or third party data, please ensure that the statement adheres to our policy

Values for all data points found in graphs can be found in the 'Supplementary data' file. Additional information or other data are available from the corresponding author upon reasonable request.

Research involving human participants, their data, or biological material

Policy information about studies with human participants or human data. See also policy information about sex, gender (identity/presentation), and sexual orientation and race, ethnicity and racism.

Reporting on sex and gender	We did not use the data involving human participants or human data.
Reporting on race, ethnicity, or other socially relevant groupings	same as above.
Population characteristics	same as above.
Recruitment	same as above.
Ethics oversight	same as above.

Note that full information on the approval of the study protocol must also be provided in the manuscript.

Field-specific reporting

Please select the one below that is the best fit for your research. If you are not sure, read the appropriate sections before making your selection.

☒ Life sciences ☐ Behavioural & social sciences ☐ Ecological, evolutionary & environmental sciences

For a reference copy of the document with all sections, see nature.com/documents/nr-reporting-summary-flat.pdf

Life sciences study design

All studies must disclose on these points even when the disclosure is negative.

Sample size	Selected as the number needed to produce a statistically significant difference.
Data exclusions	No data were excluded from the analysis.
Replication	We replicated three to six times for each experiment.
Randomization	Cells are randomly seeded.
Blinding	Blind testing is unnecessary because there is no room for arbitrary elements to influence this experiment.

Reporting for specific materials, systems and methods

We require information from authors about some types of materials, experimental systems and methods used in many studies. Here, indicate whether each material, system or method listed is relevant to your study. If you are not sure if a list item applies to your research, read the appropriate section before selecting a response.

Materials & experimental systems

n/a	Involved in the study	
☐	☒	Antibodies
☐	☒	Eukaryotic cell lines
☒	☐	Palaeontology and archaeology
☒	☐	Animals and other organisms
☒	☐	Clinical data
☒	☐	Dual use research of concern
☒	☐	Plants

Methods

n/a	Involved in the study	
☒	☐	ChIP-seq
☒	☐	Flow cytometry
☒	☐	MRI-based neuroimaging

Antibodies

Antibodies used	anti-HA and anti-GFP antibodies.
Validation	Validation data are available from the manufacturer's web site, https://www.thermofisher.com/antibody/product/GFP-Antibody-Polyclonal/A-6455 and https://ruo.mbl.co.jp/bio/dtl/A/index.html?pcd=M180-3.

Eukaryotic cell lines

Policy information about cell lines and Sex and Gender in Research

Cell line source(s)	We established in the following paper.<br>Fujii S., Kurokawa K., Inaba R., Hiramatsu N., Tago T., Nakamura Y., Nakano A., Satoh T. and Satoh A. K. Recycling endosomes are attached to trans-side of Golgi units both in Drosophila and mammalian cells. Journal of Cell Science, 133: jcs236935, 2020.
Authentication	Cell line used is not authenticated.
Mycoplasma contamination	Cell lines are not tested for mycoplasma contamination.
Commonly misidentified lines<br>(See ICLAC register)	There is no commonly misidentified lines.

Plants

Seed stocks	We did not use plants.
Novel plant genotypes	same as above.
Authentication	same as above.
